# The Effects of the Context-Dependent Codon Usage Bias on the Structure of the nsp1*α* of Porcine Reproductive and Respiratory Syndrome Virus

**DOI:** 10.1155/2014/765320

**Published:** 2014-08-03

**Authors:** Yao-zhong Ding, Ya-nan You, Dong-jie Sun, Hao-tai Chen, Yong-lu Wang, Hui-yun Chang, Li Pan, Yu-zhen Fang, Zhong-wang Zhang, Peng Zhou, Jian-liang Lv, Xin-sheng Liu, Jun-jun Shao, Fu-rong Zhao, Tong Lin, Laszlo Stipkovits, Zygmunt Pejsak, Yong-guang Zhang, Jie Zhang

**Affiliations:** ^1^State Key Laboratory of Veterinary Etiological Biology, National Foot-and-Mouth Disease Reference Laboratory, Lanzhou Veterinary Research Institute, Chinese Academy of Agricultural Sciences, Lanzhou, Gansu 730046, China; ^2^Jiangsu Co-Innovation Center for Prevention and Control of Important Animal Infectious Diseases and Zoonoses, Yangzhou, Jiangsu 225009, China; ^3^RT-Europe Research Center Ltd., Budapest, Hungary; ^4^Department of Swine Diseases, National Veterinary Research Institute, 57 Partyzantow, 24-100 Puławy, Poland

## Abstract

The information about the crystal structure of porcine reproductive and respiratory syndrome virus (PRRSV) leader protease nsp1*α* is available to analyze the roles of tRNA abundance of pigs and codon usage of the *nsp1* *α* gene in the formation of this protease. The effects of tRNA abundance of the pigs and the synonymous codon usage and the context-dependent codon bias (CDCB) of the *nsp1* *α* on shaping the specific folding units (*α*-helix, *β*-strand, and the coil) in the nsp1*α* were analyzed based on the structural information about this protease from protein data bank (PDB: 3IFU) and the *nsp1* *α* of the 191 PRRSV strains. By mapping the overall tRNA abundance along the *nsp1* *α*, we found that there is no link between the fluctuation of the overall tRNA abundance and the specific folding units in the nsp1*α*, and the low translation speed of ribosome caused by the tRNA abundance exists in the *nsp1* *α*. The strong correlation between some synonymous codon usage and the specific folding units in the nsp1*α* was found, and the phenomenon of CDCB exists in the specific folding units of the nsp1*α*. These findings provide an insight into the roles of the synonymous codon usage and CDCB in the formation of PRRSV nsp1*α* structure.

## 1. Introduction

Porcine reproductive and respiratory syndrome virus (PRRSV) is an economically important pathogen of swine. The PRRSV belongs to the order Nidovirales, family Arteriviridae, genus* Arterivirus* [1]. The PRRSV genome contains at least 9 open reading frames, including ORF1a encoding papain-like cysteine protease, ORF1b encoding RNA dependent RNA polymerase, ORFs 2–6 encoding envelop proteins, and ORF7 encoding the nucleocapsid protein [2, 3]. PRRSV strains can be divided into two distinct serotypes, namely, the North American isolate (US) and the European isolate (EU) [4–8].

The replicative enzymes of the PRRSV are encoded in ORF1a and ORF1b, which locate in the 5′ proximal three quarters of the viral genome. The two polyproteins encoded by ORF1a and ORF1b are cleaved extensively by the nonstructural protein 4 (nsp4) deriving from ORF1a, yielding a series of nonstructural proteins [9]. In particular, the nsp1 and the nsp2 proteases release themselves from the ORF1a polyprotein firstly, and the nsp1 can be further processed into two multifunctional proteases, namely, the nsp1*α* and the nsp1*β* [10, 11]. The arterivirus nsp1 region contains a tandem of papain-like autoprotease domains (PCP*α* and PCP*β*), and the arterivirus PCP*α* and PCP*β* domains were found to be active in the reticulocyte lysates and the* E. coli* systems [12, 13]. This biological feature might indicate that the active functions of PCP*α* and PCP*β* are free from the different types of the expression systems and depend on the correct folding by themselves. As for the nsp1*α*, it plays an important role in regulating the accumulation of both genome- and subgenome-length minus-strand RNA and thereby fine-tuning the relative abundance of each of viral mRNAs in the infected cells [10, 14, 15]. The correct secondary structure of the nsp1*α* is required for the biological functions of the protease. Based on the crystal structure of the nsp1*α*, it was found that this nonstructural protein has three domains, namely, the N-terminal zinc finger (ZF) domain, the papain-like cysteine protease domain, and the carboxyl-terminal extension [16]. Recently, the role of the nsp1*α* in impairing the host immune response has been reported [17]; however, little information about the relationship between synonymous codon usage and the secondary structure of the PRRSV nsp1*α* is available to date.

The synonymous codon usage and translational speed of gene play important roles in many biological functions, like translation efficiency, genetic diversity, amino acid conservation, transfer RNA abundance, coevolution of the virus and its hosts, and context-dependent codon bias (CDCB), and so forth [18–22]. The nucleotide composition of a coding sequence (CDS) is nonrandom, and the CDS nonrandomness is influenced by the preferences in the selection of synonymous codons pairing to the same amino acid (termed as the synonymous codon usage bias SCUB). The link between SCUB and specific folding unit of protein gives us a new insight into the correct formation of the secondary structure of proteins [23–26]. It is noted that mRNA sequences generally have an additional potential to carry correct structural information in the forms of SCUB, which can be involved in a single codon or a nucleotide context of the target coding sequence [27, 28]. As for SCUB, neighboring nucleotides flanking a codon regulate the usage of the specific codon from the synonymous family, termed as context-dependent codon bias (CDCB) [20, 29–31]. It has been reported that the most important nucleotide determining CDCB is the first nucleotide after a codon, termed as the *N*_1_ context [32]. Although several evidences indicate the link between SCUB and the formation of the specific folding unit of viral protein, little information about the role of CDCB in the formation of the specific folding unit is reported up to date. In this study, we employed the structural information about the nsp1*α* of PRRSV and several simple formulas to analyze the relationship between the CDCB of the PRRSV nsp1*α* gene and the protease.

## 2. Materials and Methods

### 2.1. Information of PRRSV Gene and Structure of the* nsp1* *α*

The 191 coding sequences of PRRSV containing the* nsp1* *α* gene were downloaded from the National Center for Biotechnology Information (NCBI) (http://www.ncbi.nlm.nih.gov/Genbank/) and the accession numbers of the sequences were listed in Table S1 available online at http://dx.doi.org/10.1155/2014/765320. To investigate SCUB of the* nsp1* *α* , the related genes were obtained from these 191 coding sequences by the multiple sequence alignments performed with the Clustal W (1.7) computer programs [33]. The information about the secondary structure of the PRRSV nsp1*α* was obtained from protein data bank (PDB: 3IFU).

### 2.2. Analysis of the Overall tRNA Abundance of Each Codon Position along the nsp1*α* Gene

To identify the translation selection caused by the various tRNA copy numbers (reflecting tRNA abundance) of the pigs (http://gtrnadb.ucsc.edu/) at each codon position in the PRRSV* nsp1* *α* , we devised an index (*C* value) representing the overall tRNA abundance for a particular codon position in a target gene. Consider

(1) $$\begin{array}{c} C=\sqrt[{n}]{\overset{n}{\underset{1}{\prod }}\left(\frac{Wij}{Wj}\right)}, \end{array}$$

where *C* value indicates the overall tRNA abundance for a particular codon position in the target gene, *W*_*ij*_ represents the tRNA copy numbers of a synonymous codon (*i*) for the corresponding amino acid (*j*), *W*_*j*_ represents the optimal tRNA copy numbers of a synonymous codon for the same amino acid, and *n* means the number of the interesting gene. The *C* value ranges from 0 to 1.0. The *C* value less than 0.3 for a codon position represents low tRNA abundance, and the *C* value more than 0.7 for a codon position represents high tRNA abundance.

### 2.3. Estimation of the Relationship between the Synonymous Codon Usage Bias and the Secondary Structure of the nsp1*α*

Based on the alignment between the amino acid sequences of the PRRSV (PDB: 3IFU) and the 191 nsp1*α* genes involved in this study, we can locate the different folding units in the target protein. We devised the formula for the *P* value based on the previous research which analyzed the relationship between the codon usage bias and the structure of the target protein [25]. Consider

(2) $$\begin{array}{c} P=\ln\frac{f_{\text{obs}}}{f_{\exp}},\\ f_{\text{obs}}=\frac{N_{\left(i,\text{sec-}k\right)}}{N_{\left(k\right)}},\\ f_{\exp}=\frac{\sum N_{\left(i,\text{sec-}j\right)}}{N_{\text{total}}}, \end{array}$$

where *N*_(*i*,sec-*k*)_ represents the amount of a specific synonymous codon for the corresponding amino acid in a specific folding unit (the *α*-helix, the *β*-strand, or the coil) of protein; sec-*k* represents the corresponding amino acid in a specific secondary unit; *N*_(*k*)_ represents the amount of the amino acid in the corresponding folding unit. In addition, ∑*N*_(*i*,sec-*j*)_ represents the total number of amino acids in a specific folding unit; sec-*j* contains the three kinds of folding unit, namely, *α*-helix, *β*-strand, and the coil; *N*_total_ represents the total number of codons in the target genes. When the *P* value is more than zero, the corresponding synonymous codon (*i*) owns a potential to be selected in a specific folding unit. When the *P* value is less than zero, the synonymous codon (*i*) has no tendency to be chosen in a specific folding unit. Furthermore, we defined that when the *P* value is more than 0.1, the synonymous codon has a strong ability to exist in the specific folding unit; on the contrary, when the *P* value is less than −0.1, the synonymous codon has a strong tendency to avoid the specific folding unit.

### 2.4. Calculation of the Relative Abundance of Codons with Context

With the purpose to estimate the synonymous codons playing an important role in the formation of the specific folding units, codons having a significant tendency to exist in the specific folding unit of the PRRSV* nsp1* *α* were analyzed by the formula for the relative abundance of codons with context. Berg and Silva [32] defined that the context *N*_1_ represents the first nucleotide after the target codon. Following this notation, we defined that the context _1_ *N* represents the last nucleotide before the target codon. We devised a formula calculating *R* value for the context *N*_1_(*xyz*~*n*) and the context _1_ *N*(*n*~*xyz*) depending on the formula previously reported [20, 34]. Consider

(3) $$\begin{array}{c} R\left(xyz\sim n\right)=\frac{F\left(xyz\sim n\right)}{F\left(xyz\right)F\left(n\right)},\\ R\left(n\sim xyz\right)=\frac{F\left(n\sim xyz\right)}{F\left(xyz\right)F\left(n\right)}, \end{array}$$

where *F*(*xyz*) is the frequency of the codon *xyz* and *F*(*n*) is the frequency of nucleotide *n* in the *N*_1_ or _1_ *N* context. *F*(*xyz*~*n*) and *F*(*n*~*xyz*) are the frequency of a codon with the *n* context. It is noted that *x*, *y*, *z*, and *n* are the nucleotides (*a*, *u*, *g*,  or  *c*) and the codon is composed of *xyz*. Here and elsewhere the tilde character (~) separates codons (italic) or oligonucleotides (nonunderlined) from their mononucleotide context.

### 2.5. Calculation of the Relative Abundance of Mononucleotide and Dinucleotides in the* nsp1* *α* Gene

To investigate whether the *N*_1_ and _1_ *N* contexts are shaped by randomness or not, we calculated the frequencies of each nucleotide *F*(*n*) and dinucleotide *F*(*xy*), where *n*, *x*, *y*, and *z* are each one of the four nucleotides (*a*, *u*, *c*, and *g*). Then we calculated the relative abundances (*r* value) of the mononucleotide and dinucleotides with a single nucleotide context: *r*(*n*~*x*) = *F*(*n*~*x*)/[*F*(*n*)*F*(*x*)], for mononucleotide *x* with context *n*; *r*(*xy*~*n*) = *F*(*xy*~*n*)/[*F*(*xy*)*F*(*n*)], for dinucleotide*xy* with context *n*.

### 2.6. Statistic Analysis

One-way analysis of variance, namely, one-way ANOVA, is a technique used to compare means of two or more samples. In this study, the ANOVA test is applied for identifying whether the overall tRNA abundance of positions of a specific folding unit is different from other specific folding units or not. In addition, the ANOVA test is also employed to estimate whether the frequencies of codon usage in a specific folding unit are different from other specific folding units or not. This statistic analysis is carried out by the software SPSS 11.5.

## 3. Results

### 3.1. The Overall tRNA Abundance for Each Codon Position of the* nsp1* *α* Gene

**Figure 1:**
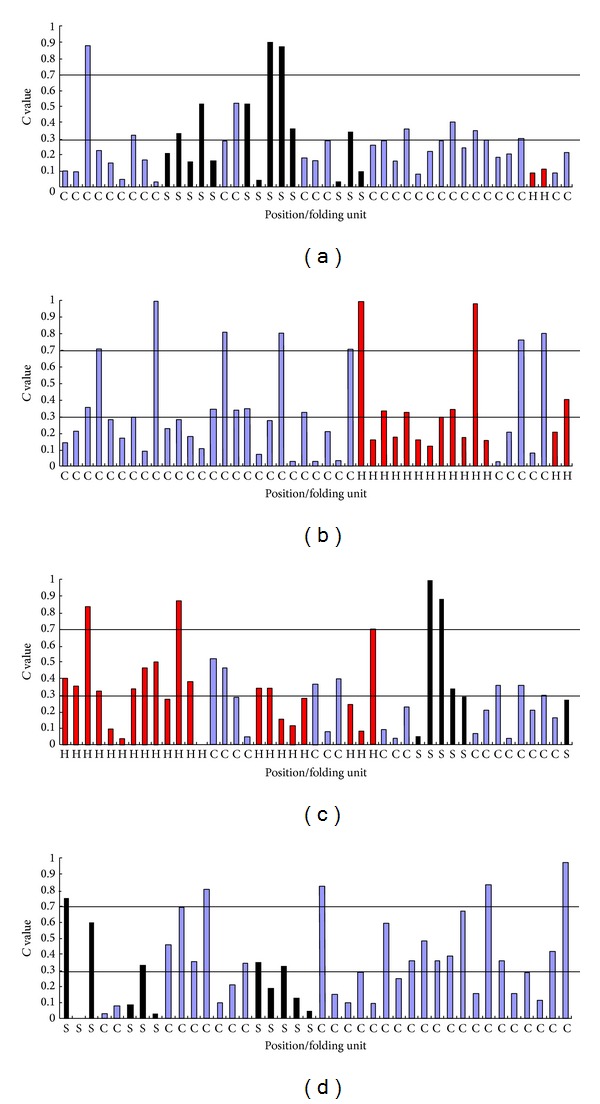
The overall tRNA abundance for each codon position of the PRRSV* nsp1* *α* gene. The black bar corresponds to the *β*-strand region in the* nsp1* *α*; the blue bar corresponds to the coil region in the* nsp1* *α*; the red bar corresponds to helix region in the* nsp1* *α* . (a) The codon positions range from the first position to the 45th position of the* nsp1* *α* . (b) The codon positions range from the 46th position to the 90th position of the* nsp1* *α* . (c) The codon positions range from the 91th position of the* nsp1* *α* . (d) The codon positions range from the 136th to the 175th position of the* nsp1* *α* .

Based on the *C* values, the tRNA abundance for each codon position along the PRRSV nsp1*α* gene was mapped. The translation speed for the synthesis of the nsp1*α* is not stable in the pigs (Figure 1). The codon positions with the *C* values much less than 0.30 have a tendency to cluster in* nsp1* *α* gene, including the positions 4–6, 8–10, 22–25, 27–30, 32–34, 38–40, 42–47, 50–53, 55–58, 68–70, 77–79, 83–85, 110–112, 119–122, 126–128, 139–141, 157–160, and 171–173. However, the codon positions with the *C* values much greater than 0.70 have few chances to cluster in* nsp1* *α* gene which is translated in the pigs. Due to most codon positions with *C* values much less than 0.70 existing in the target gene, these positions within the* nsp1* *α* might reduce the translation rate of this protein when the* nsp1* *α* was scanned by the ribosomes in pig cells. It is noted that there are no significant differences (*P* > 0.05) of the overall tRNA abundance for the codon positions in the regions of the three specific folding units of the nsp1*α*. This result suggests that the fluctuation of the overall tRNA abundance pairing to each codon position along this* nsp1* *α* might not regulate the formation of the specific folding units but decrease the scanning speed of ribosomes in the pig cells.

### 3.2. The Relationship between the Synonymous Codon Usage Bias and the Structure of the* nsp1* *α*

Based on the *P* values for the synonymous codons which are involved in the formation of the specific folding units in the nsp1*α*, we found the link between SCUD and the specific folding unit (*P* = 2.75 × 10^−11^). In detail, the synonymous codons have a strong propensity toward shaping the *α*-helix unit, including AUC for Ile, GUA for Val, AGC for Ser, AAG for Lys, and AUG for Met (Table 1). Turning to the effects of SCUB on shaping the *β*-strand unit, there are UUA for Leu, AUA for Ile, GUG for Val, UCA and AGU for Ser, ACA for Thr, UAC for Tyr, CGC for Arg, and two synonymous codons for His (Table 1). It is interesting that there are no codons which have a strong tendency to exist in the coil of the nsp1*α* (Table 1). As for the codons which have a strong tendency (*P* value > 1.0) to exist in the nsp1*α*, all of them strongly tend to exist only in the *α*-helix or the *β*-strand of this protein.

### 3.3. The Relative Abundance of the Codon with *N*_1_ Context in the* nsp1* *α* Gene

As for the codons which have a strong tendency to exist in the specific folding unit of the nsp1*α*, their *R* values, the relative abundance of codons with *N*_1_ contexts, were calculated from the 191 nsp1*α* genes (Table 2). The data show that the occurrence of the codon with *N*_1_ context or _1_ *N* context is not random, and many codons with *N*_1_ context or _1_ *N* context have a strong tendency to exist in the specific folding units of the nsp1*α*. Based on the data of SCUB in the specific folding units (Table 1), the corresponding codon with *N*_1_ context was found to have a trend to exist in the specific folding unit of the nsp1*α*. In detail, the codons with *N*_1_ context or _1_ *N* context (GUA~A, AGC~A, AAG~C, AGA~C, A~AUA, U~AGC, U~AAG, C~AAG, G~AGA, and U~AUG) have an obvious trend to exist in the helix unit of the nsp1*α*. Some codons with *N*_1_ context or _1_ *N* context have a strong tendency to exist in the *β*-strand of the nsp1*α*, including UUA~A, AUA~G, GUG~U, UCA~C, AGU~G, ACA~C, UAC~U, UAC~C, CAU~G, CAC~G, CGC~U, G~UUA, U~AUA, U~GUG, G~GUG, U~UCA, C~AGU, C~ACA, C~UAC, G~UAC, U~CAU, U~CAC, and U~CGC.

In order to identify the roles of nucleotide compositions (dinucleotide with *N*_1_ context and mononucleotide with *N*_1_ context) in shaping the codon with *N*_1_ context or _1_ *N* context, the *R* values for these interesting codons with *N*_1_ context or _1_ *N* context which have a strong tendency to exist in the helix or the *β*-strand were compared with the *r* values for the dinucleotide/mononucleotide with *N*_1_ context (Tables 3 and 4). The *R* value for the target codon with *N*_1_ context is higher than the corresponding dinucleotide/mononucleotide with *N*_1_ context or _1_ *N* context. For example, as for GUA which tends to exist in the helix of the nsp1*α* gene, GUA~A has a tendency to exist in the helix unit, because the *R* value (1.4751) of GUA~A for the helix unit is higher than the *R* value for GUA~A for the *β*-strand and the coil (Table 2) and higher than the *r* value for UA~A and the *r* value for A~A (Tables 3 and 4). As for UUA which tends to exist in the *β*-strand of this gene, UUA~A has a tendency to exist in the strand unit, because the *R* value (4.9268) for UUA~A is higher than the *R* values for UUA~A of the helix and the coil and higher than the *r* values for UA~A and A~A (Tables 3 and 4). As for AGC which tends to exist in the helix of this gene, U~AGC has a tendency to exist in the helix rather than in the strand or the coil, because the *R* value (5.9004) for U~AGC is higher than the *R* value of U~AGC of the strand and the coil and higher than the *r* values for U~AG and U~A (Tables 3 and 4). As for UUA which tends to exist in the strand of this gene, G~UUA has a tendency to exist in the strand unit, because the *R* value (3.7019) for G~UUA of the strand unit is higher than the *R* values for G~UUA of the helix and the coil and higher than *r* values for G~UU and G~U (Tables 3 and 4). Based on the standard mentioned above, GUA~A, AGC~A, AAG~C, AGA~C, A~GUA, U~AGC, U~AAG, C~AAG, G~AGA, and U~AUG have a strong trend to exist in the helix of PRRSV nsp1*α* gene and UUA~A, AUA~G, GUG~U, UCA~C, ACA~C, UAC~U, UAC~C, CAU~G, CGC~U, G~UUA, U~GUG, U~UCA, C~AGU, C~ACA, G~UAC, U~CAU, and U~CGC have a strong tendency to exist in the *β*-strand of the nsp1*α* gene.

## 4. Discussion

In this study, we have mapped the fluctuation of the overall tRNA abundance for each codon position along the PRRSV nsp1*α* gene and estimated the correlation between the synonymous codon usage and different folding units of the nsp1*α*. The performance of mapping the fluctuation of the overall tRNA abundance for each codon position along the target gene likely reflects the translation speed of ribosomes scanning caused by the tRNA abundance of the pigs to some degree, since the tRNA abundance plays an important role in the ribosome scanning along the target coding sequence [35, 36]. The previous report showed that the *α*-helix is preferentially coded by translationally fast mRNA regions while the slow segments often encode *β*-strands and coil regions [37]. In the study, no linkage between the fluctuation of the overall tRNA abundance pairing to the codon positions along the nsp1*α* gene and the specific folding units might suggest that the process of translation fine-tunes is not performed by variation of translation speed for each codon position along the* nsp1* *α* . The fine-tuning* in vivo* protein folding exists in the gene, and this regularity is largely believed to occur in a cotranslational process [38]. However, the PRRSV nsp1*α* derives from the posttranslational processing of the pp1a [10, 39]. The process of the cleavage of the nsp1*α* from the pp1a polyprotein of PRRSV performed by the posttranslation might be free from the fluctuation of tRNA abundance pairing to the each codon position along the nsp1*α* gene. As for the ribosomes scanning the nsp1*α* gene, there is no significant link between the fluctuation of the overall tRNA abundance and the specific folding units, and the translation elongation rate of this gene is not high. These results suggest that the low tRNA abundance controls the ribosomal traffic along the translated message to achieve the effective synthesized product of the PRRSV pp1a. The low translational elongation at the translation beginning step directs the target gene to generate the corresponding protein effectively [40].

Turning to the role of the synonymous codon usage in the formation of the specific units of the nsp1*α*, there is significant relationship between the synonymous codon usage bias and the specific folding units in the target protein. The synonymous codons assist messenger RNA to carry the information of the specific folding units, and a single codon or a contiguous nucleotide region plays roles in shaping the specific folding units [24, 25, 41, 42]. As for the PRRSV* nsp1* *α* , there is no synonymous codon which tends to exist in coil unit. However, many synonymous codons exist in the *α*-helix and *β*-strands regions of this gene, and no synonymous codon has a strong tendency to be selected by both the *α*-helix and the *β*-strands in the PRRSV* nsp1* *α* simultaneously. These results indicated that SCUB might play roles in shaping this protease with natural properties for the life-cycle of PRRSV. SCUB for formation of the specific folding units of the PRRSV nsp1*α* is influenced by the natural selection. As an example of the role for natural selection, the expressivity of genes is an important factor in shaping SCUB, both for prokaryotic and for eukaryotic organisms [18, 22, 43, 44]. Although the link between the SCUB and the formation of the specific folding units was reported [25, 35, 37, 38, 45], the role of CDCB in formation of specific folding units is not clear. In this study, we found that CDCB plays a role in the formation of specific folding units in the PRRSV nsp1*α*. The synonymous usage bias and CDCB, which play important roles in achieving accuracy and efficiency in protein synthesis, are particular manifestations of coding sequence nonrandomness [23, 46, 47]. Spatial interaction of ribosomal proteins with codon-anticodon RNA pairs inside the A and P sites of the ribosome could be preferable for particular codons with context [20, 48].

## Supplementary Material

Table 1: The accession number of the 191 strains of PRRSV

## Figures and Tables

**Table 1:** The relationship between the synonymous codon usage and the formation of the specific secondary structure unit.

Amino acid	Codon	^a^ *P* value	^b^ *P* value	^c^ *P* value
Phe	UUU	0.40	0.07	−0.21
Phe	UUC	−0.92	−0.40	0.26
Leu	UUA	−*1.07 *	**1.47**	*−1.22 *
Leu	UUG	−0.16	−0.53	0.15
Leu	CUU	0.68	^ d^—	−0.06
Leu	CUC	*−5.53 *	*−5.32 *	0.48
Leu	CUA	0.17	*−1.37 *	0.13
Leu	CUG	0.00	*−4.15 *	0.24
Ile	AUU	0.99	0.92	*−5.46 *
Ile	AUC	**1.50**	*−1.17 *	^ d^—
Ile	AUA	0.69	**1.12**	*−2.46 *
Val	GUU	0.58	0.28	−0.45
Val	GUC	0.32	0.20	−0.21
Val	GUA	**1.27**	0.02	*−2.17 *
Val	GUG	−0.52	**1.21**	−0.73
Ser	UCU	0.76	^ d^—	−0.12
Ser	UCC	*−4.45 *	*−3.14 *	0.47
Ser	UCA	^ d^—	**1.76**	^ d^—
Ser	UCG	^ d^—	^ d^—	^ d^—
Ser	AGU	*−4.79 *	**1.35**	−0.61
Ser	AGC	**1.24**	−0.25	*−1.53 *
Pro	CCU	−0.48	*−2.90 *	0.33
Pro	CCC	*−2.85 *	−0.28	0.33
Pro	CCA	−0.32	*−4.40 *	0.31
Pro	CCG	−0.23	*−2.69 *	0.28
Thr	ACU	−0.08	0.58	−0.21
Thr	ACC	−0.57	0.53	−0.05
Thr	ACA	0.58	**1.28**	*−5.24 *
Thr	ACG	*−4.40 *	^ d^—	0.48
Ala	GCU	0.55	*−2.56 *	0.00
Ala	GCC	−0.07	0.10	−0.01
Ala	GCA	0.78	0.16	−0.61
Ala	GCG	0.76	1.15	*−4.18 *
Tyr	UAU	0.95	*−1.60 *	−0.39
Tyr	UAC	0.21	**1.37**	*−2.22 *
His	CAU	*−4.21 *	**1.75**	*−4.18 *
His	CAC	^ d^—	**1.75**	*−3.81 *
Gln	CAA	0.62	−0.82	−0.15
Gln	CAG	*−2.74 *	0.63	0.07
Asn	AAU	*−4.47 *	−0.58	0.38
Asn	AAC	^ d^—	0.18	0.25
Lys	AAA	−0.90	^ d^—	0.39
Lys	AAG	**1.18**	^ d^—	−0.69
Asp	GAU	^ d^—	*−3.17 *	0.48
Asp	GAC	^ d^—	^ d^—	0.48
Glu	GAA	0.93	^ d^—	−0.28
Glu	GAG	*−1.15 *	*−5.28 *	0.41
Cys	UGU	^ d^—	*−4.75 *	0.48
Cys	UGC	*−5.41 *	0.04	0.28
Arg	CGU	*−1.82 *	0.28	0.18
Arg	CGC	^ d^—	**1.52**	*−1.03 *
Arg	CGA	0.83	*−2.13 *	−0.22
Arg	CGG	−0.04	0.16	−0.04
Arg	AGA	**1.37**	*−4.30 *	*−1.32 *
Arg	AGG	^ d^—	0.76	0.03
Gly	GGU	0.52	^ d^—	0.04
Gly	GGC	−0.34	*−4.61 *	0.32
Gly	GGA	*−4.76 *	*−4.55 *	0.48
Gly	GGG	^ d^—	*−4.62 *	0.48
Met	AUG	**1.01**	−0.35	−0.72
Trp	UGG	0.45	0.66	−0.61

^a^represents*α*-helix.

^b^represents*β*-strand.

^c^represents The coil.

^d^represents The corresponding codon is not selected in the specific secondary structure unit.

Italic indicates that the corresponding codon has a weak bias to be selected in a specific secondary structure unit.

Bold indicates that the corresponding codon has a tendency to be selected in a specific secondary structure unit.

**Table 2:** Relative abundance of codons with *N*_1_ context or _1_ *N* context in the PRRSV *nsp1* *α* gene.

Codon-context (*xyz*-*n*)	*R* value^1^	*R* value^2^	*R* value^3^	Codon-context (*xyz*-*n*)	*R* value^1^	*R* value^2^	*R* value^3^
UUA~A	0.0000	4.9268	0.3345	A~UUA	0.0000	0.0401	3.6799
UUA~U	0.2911	0.0000	0.0000	U~UUA	0.1456	0.0617	0.2945
UUA~C	2.8120	0.0000	0.0000	C~UUA	2.9124	0.0000	0.0000
UUA~G	0.0000	0.0000	3.7713	G~UUA	0.0000	3.7019	0.0000
AUC~A	0.0000	0.0000	0.0000	A~AUC	0.0000	0.0000	2.4430
AUC~U	0.0000	0.0000	3.1392	U~AUC	0.0000	0.2233	0.0116
AUC~C	0.0000	0.0000	0.0000	C~AUC	0.0000	3.4849	0.0439
AUC~G	0.0000	3.7945	0.4601	G~AUC	0.0000	0.0000	1.5563
AUA~A	0.0000	0.0000	0.0000	A~AUA	0.0000	0.0000	0.0000
AUA~U	0.0000	0.0000	0.0000	U~AUA	4.3672	3.4158	2.2091
AUA~C	3.0128	0.0000	0.5557	C~AUA	0.0000	0.3703	0.0000
AUA~G	0.0000	3.7945	3.5998	G~AUA	0.0000	0.0000	1.5428
GUA~A	1.4751	0.0000	0.0934	A~GUA	2.9502	3.2845	0.0000
GUA~U	1.0918	3.7954	0.0822	U~GUA	0.0000	0.0000	0.0000
GUA~C	0.7532	0.0000	4.2386	C~GUA	0.7532	1.2342	0.0000
GUA~G	0.9274	0.0000	0.0000	G~GUA	0.9274	0.0000	4.1141
GUG~A	0.0510	0.0000	0.0000	A~GUG	1.4283	0.0000	0.3526
GUG~U	0.2014	2.7198	0.0000	U~GUG	0.0629	1.8132	0.0239
GUG~C	1.4413	0.1874	4.4454	C~GUG	0.0174	0.4872	0.0000
GUG~G	1.7318	0.8833	0.0000	G~GUG	2.7367	1.4824	3.7249
UCA~A	0.0000	0.0000	0.0000	A~UCA	0.0000	0.0000	0.0000
UCA~U	0.0000	0.0000	0.0000	U~UCA	0.0000	3.7954	0.0000
UCA~C	0.0000	3.7027	0.0000	C~UCA	0.0000	0.0000	0.0000
UCA~G	0.0000	0.0000	0.0000	G~UCA	0.0000	0.0000	0.0000
AGU~A	0.0000	0.0000	0.0000	A~AGU	0.0000	0.0000	0.0000
AGU~U	0.0000	0.0000	0.0000	U~AGU	2.4694	0.0000	0.0000
AGU~C	0.0473	0.0000	0.0000	C~AGU	1.2619	3.7027	4.4454
AGU~G	3.6513	3.7945	4.1141	G~AGU	0.0583	0.0000	0.0000
AGC~A	2.9502	0.0000	0.0000	A~AGC	0.0000	0.0000	0.0000
AGC~U	0.0000	0.0000	0.0000	U~AGC	5.9004	0.0000	0.0000
AGC~C	0.0000	0.0000	0.0000	C~AGC	0.0000	3.7027	0.0000
AGC~G	1.8548	3.7945	0.0000	G~AGC	0.0000	0.0000	0.0000
ACA~A	0.0000	0.0000	0.0000	A~ACA	0.0000	0.0000	0.0000
ACA~U	0.0000	0.0000	0.0000	U~ACA	4.3672	0.0000	3.4431
ACA~C	0.0000	3.7027	0.0383	C~ACA	0.0000	3.7027	0.1150
ACA~G	3.7096	0.0000	4.0786	G~ACA	0.0000	0.0000	0.0000
UAC~A	5.6595	0.0000	3.9513	A~UAC	0.0000	0.0502	0.0000
UAC~U	0.0000	1.4455	0.0000	U~UAC	1.2478	0.8812	1.4434
UAC~C	0.1230	1.4253	0.0000	C~UAC	2.1520	1.3876	2.6300
UAC~G	0.0000	0.8887	0.0646	G~UAC	0.0000	1.4529	0.0000
CAU~A	0.0000	0.0000	0.0000	A~CAU	3.9336	0.0000	0.0000
CAU~U	0.0000	0.0000	0.0000	U~CAU	0.0000	3.7043	0.0000
CAU~C	1.0043	0.0000	0.0000	C~CAU	0.0000	0.0889	4.4454
CAU~G	2.4730	3.7945	4.1141	G~CAU	1.2365	0.0000	0.0000
CAC~A	0.0000	0.0000	0.0000	A~CAC	0.0000	0.0000	0.0000
CAC~U	0.0000	0.2138	0.0000	U~CAC	4.3672	3.5816	0.0000
CAC~C	0.0000	0.0000	0.0000	C~CAC	0.0000	0.2086	0.0000
CAC~G	3.7096	3.5807	0.0000	G~CAC	0.0000	0.0000	0.0000
AAG~A	0.0000	0.0000	3.4986	A~AAG	0.0000	0.0000	3.3416
AAG~U	0.0546	0.0000	0.0000	U~AAG	1.4739	0.0000	0.0000
AAG~C	2.9751	0.0000	0.0000	C~AAG	1.9960	0.0000	0.0000
AAG~G	0.0000	0.0000	0.5286	G~AAG	0.0000	0.0000	0.6895
CGC~A	1.3112	0.0000	0.0000	A~CGC	2.8704	0.0000	0.0000
CGC~U	0.1213	3.7954	0.0000	U~CGC	0.1180	2.3721	0.0000
CGC~C	0.0837	0.0000	0.0000	C~CGC	1.3843	1.3885	0.0000
CGC~G	2.6791	0.0000	0.0000	G~CGC	0.1003	0.0000	0.0000
AGA~A	0.0000	0.0000	1.9453	A~AGA	0.0831	0.0000	2.0578
AGA~U	0.1230	0.0000	0.1188	U~AGA	0.6766	0.0000	0.0099
AGA~C	2.8855	0.0000	0.0249	C~AGA	0.1273	0.0000	2.1293
AGA~G	0.0522	0.0000	1.9591	G~AGA	2.9259	0.0000	0.0230
AUG~A	0.0000	0.0000	2.1721	A~AUG	0.0000	0.0000	3.9812
AUG~U	0.0000	0.0000	0.0000	U~AUG	4.1823	3.6955	0.0000
AUG~C	0.0000	0.0000	0.0000	C~AUG	0.1275	0.0974	0.0000
AUG~G	0.0000	3.7945	1.8881	G~AUG	0.0000	0.0000	0.0340

^1^represents The relative abundance of codons with *N*_1_ context in the helix unit of the PRRSV *nsp1* *α* .

^2^represents The relative abundance of codons with *N*_1_ context in the *β*-strand unit of the PRRSV *nsp1* *α* .

^3^represents The relative abundance of codons with *N*_1_ context in the coil unit of the PRRSV* nsp1* *α* .

**Table 3:** Relative abundance of dinucleotides with *N*_1_ context or _1_ *N* context in the PRRSV* nsp1* *α* gene.

Dinucleotides with *N*_1_ context (*xy*~*n*)	*r* value	Dinucleotides with _1_ *N* context (*n*~*xy*)	*r* value
UC~A	0.6130	A~AU	1.4456
UC~U	1.3115	U~AU	0.8552
UC~C	1.2805	C~AU	0.6485
UC~G	0.5669	G~AU	1.0047
UA~A	0.6177	A~GU	1.2963
UA~U	1.0269	U~GU	1.1651
UA~C	1.7402	C~GU	0.5072
UA~G	0.4351	G~GU	1.1064
UG~A	1.2060	A~UC	0.5880
UG~U	0.8195	U~UC	1.0330
UG~C	1.0921	C~UC	1.1230
UG~G	0.8019	G~UC	1.0286
CA~A	1.8155	A~AG	1.2415
CA~U	0.4556	U~AG	0.2825
CA~C	0.9184	C~AG	0.9946
CA~G	0.8963	G~AG	1.5135
GU~A	0.3165	A~AC	1.5521
GU~U	0.9679	U~AC	0.9913
GU~C	0.8485	C~AC	0.8942
GU~G	1.5896	G~AC	0.6067
GC~A	1.2887	A~UA	0.6720
GC~U	1.0849	U~UA	0.9776
GC~C	1.3411	C~UA	1.6741
GC~G	0.3298	G~UA	0.5155
CA~A	1.8155	A~CA	0.9721
CA~U	0.4556	U~CA	0.4819
CA~C	0.9184	C~CA	1.3762
CA~G	0.8963	G~CA	1.0994
AC~A	0.9465	A~AA	1.1734
AC~U	1.2243	U~AA	0.3547
AC~C	0.8848	C~AA	1.7821
AC~G	0.9524	G~AA	1.4708
AU~A	0.5596	A~CG	1.6312
AU~U	0.9124	U~CG	0.7431
AU~C	0.6579	C~CG	1.2830
AU~G	1.7819	G~CG	0.4693
AG~A	1.0440	A~UU	0.8843
AG~U	1.3708	U~UU	1.5257
AG~C	0.7761	C~UU	1.2791
AG~G	0.8164	G~UU	1.2724
GC~A	1.2887		
GC~U	1.0849		
GC~C	1.3411		
GC~G	0.3298		

**Table 4:** Relative abundance of mononucleotide with *N*_1_ context in PRRSV* nsp1* *α* gene.

Mononucleotide with N_1_ context	*r*(*x*~*n*)
A~A	1.0664
A~U	0.7354
A~C	1.0751
A~G	0.9433
U~A	0.6124
U~U	0.7588
U~C	0.8228
U~G	1.4182
C~A	1.0468
C~U	1.1225
C~C	0.8788
C~G	0.6277
G~A	1.0557
G~U	0.9975
G~C	0.8930
G~G	0.7782
