# Supplementary material for: The Effects of the Context-Dependent Codon Usage Bias on the Structure of the nsp1α of Porcine Reproductive and Respiratory Syndrome Virus
Source: Biomed Res Int. 2014 Aug 3;2014:765320. doi: 10.1155/2014/765320 (PMC4137607; doi:10.1155/2014/765320)
Supplement: Supplementary file 1 — Table 1: The accession number of the 191 strains of PRRSV [file 765320.f1.docx]

TABLE S 1The accession number of the 191 strains of PRRSV

| Accession No. | Accession No. | Accession No. | Accession No. | Accession No. |
| --- | --- | --- | --- | --- |
| AF046869 | EF517962 | EU360130 | FJ889129 | HQ416720 |
| AB288356 | EF532801 | EU624117 | FJ895329 | HQ699067 |
| AF066183 | EF532802 | EU678352 | FJ899592 | HQ843178 |
| AF159149 | EF532803 | EU708726 | GQ330474 | HQ843179 |
| AF176348 | EF532804 | EU807840 | GQ351601 | HQ843180 |
| AF184212 | EF532805 | EU825723 | GQ359108 | HQ843181 |
| AF303354 | EF532806 | EU860248 | GQ374441 | JF268672 |
| AF303355 | EF532807 | EU860249 | GQ374442 | JF268673 |
| AF303356 | EF532808 | EU864231 | GQ475526 | JF268674 |
| AF303357 | EF532809 | EU864232 | GQ499193 | JF268675 |
| AF325691 | EF532810 | EU864233 | GQ499194 | JF268676 |
| AF331831 | EF532811 | EU880431 | GQ499195 | JF268677 |
| AF494042 | EF532812 | EU880432 | GQ499196 | JF268678 |
| AY032626 | EF532813 | EU880433 | GQ857656 | JF268679 |
| AY150312 | EF532814 | EU880434 | GU143913 | JF268680 |
| AY150564 | EF532815 | EU880435 | GU168567 | JF268681 |
| AY262352 | EF532816 | EU880436 | GU168568 | JF268682 |
| AY424271 | EF532817 | EU880437 | GU168569 | JF268683 |
| AY457635 | EF532818 | EU880438 | GU169411 | JF268684 |
| AY545985 | EF532819 | EU880439 | GU232735 | JF748717 |
| AY585241 | EF535999 | EU880440 | GU232736 | JF748718 |
| AY612613 | EF536000 | EU880441 | GU232737 | JF796180 |
| DQ056373 | EF536001 | EU880442 | GU232738 | JF800911 |
| DQ176019 | EF536002 | EU880443 | GU269541 | JN256115 |
| DQ176020 | EF536003 | EU939312 | GU454850 | JN387271 |
| DQ176021 | EF635006 | FJ175687 | GU461292 | JN387272 |
| DQ217415 | EF641008 | FJ175688 | HM011104 | JN387273 |
| DQ459471 | EU097706 | FJ175689 | HM016158 | JN387274 |
| DQ473474 | EU097707 | FJ393456 | HM016159 | JN626287 |
| DQ779791 | EU106888 | FJ393457 | HM189676 | JN662424 |
| DQ988080 | EU109502 | FJ393458 | HM214913 | U87392 |
| EF075945 | EU109503 | FJ393459 | HM214914 |  |
| EF112445 | EU144079 | FJ394029 | HM214915 |  |
| EF112446 | EU187484 | FJ536165 | HM853673 |  |
| EF112447 | EU200961 | FJ548851 | HQ233604 |  |
| EF153486 | EU200962 | FJ548852 | HQ233605 |  |
| EF484031 | EU236259 | FJ548853 | HQ315835 |  |
| EF484033 | EU262603 | FJ548854 | HQ315836 |  |
| EF488048 | EU360128 | FJ548855 | HQ315837 |  |
| EF488739 | EU360129 | FJ797690 | HQ401282 |  |
